# Antiviral Effects and Underlying Mechanisms of Probiotics as Promising Antivirals

**DOI:** 10.3389/fcimb.2022.928050

**Published:** 2022-06-06

**Authors:** Yanjin Wang, Assad Moon, Jingshan Huang, Yuan Sun, Hua-Ji Qiu

**Affiliations:** State Key Laboratory of Veterinary Biotechnology, Harbin Veterinary Research Institute, Chinese Academy of Agricultural Sciences, Harbin, China

**Keywords:** probiotics, viral infections, antiviral effects, antiviral mechanisms, novel antivirals

## Abstract

Probiotics exert a variety of beneficial effects, including maintaining homeostasis and the balance of intestinal microorganisms, activating the immune system, and regulating immune responses. Due to the beneficial effects of probiotics, a wide range of probiotics have been developed as probiotic agents for animal and human health. Viral diseases cause serious economic losses to the livestock every year and remain a great challenge for animals. Moreover, strategies for the prevention and control of viral diseases are limited. Viruses enter the host through the skin and mucosal surface, in which are colonized by hundreds of millions of microorganisms. The antiviral effects of probiotics have been proved, including modulation of chemical, microbial, physical, and immune barriers through various probiotics, probiotic metabolites, and host signaling pathways. It is of great significance yet far from enough to elucidate the antiviral mechanisms of probiotics. The major interest of this review is to discuss the antiviral effects and underlying mechanisms of probiotics and to provide targets for the development of novel antivirals.

## Introduction

Humans and animals are colonized by hundreds of millions of microorganisms, which are far more than the number of host cells, and some argue that the number of microorganisms is comparable to host cells (Egert and Simmering, 2016). A vast majority of these microorganisms coinhabit within the gastrointestinal tract (Sender et al., 2016; Heintz-Buschart and Wilmes, 2018; Mishra et al., 2021). Probiotics are essential to maintain the balance of intestinal microorganisms. The Food and Agriculture Organization (FAO) and the World Health Organization (WHO) define probiotics as probiotics are live microorganisms, which when administered in adequate amounts confer health benefits on the host (WHO/FAO, 2002; Drago et al., 2010; Kahouli et al., 2013). Probiotics are known for their beneficial effects, mainly including maintaining the balance of intestinal microorganisms, activating the immune system, and regulating immune response (Hao et al., 2015; Gasbarrini et al., 2016; Kim et al., 2019). Due to their beneficial effects, a variety of probiotics have been widely used for promoting animal and human health in recent years (Kim et al., 2019; Teame et al., 2020; Rad et al., 2021). Several species of probiotics and their effects as probiotic agents have been evaluated. *Lactobacillus*, *Bifidobacterium*, and yeast are widely used (Al-Ghazzewi and Tester, 2016; Wieers et al., 2020; Di Pierro and Pane, 2021; Giannakou et al., 2021).

Viral diseases remain a great challenge for humans and animals (VanderWaal and Deen, 2018). Although vaccination is the most important option to prevent viral infections, differences between evolving epidemics and vaccines available make vaccination less effective. Moreover, there are no available vaccines for emerging or re-emerging viruses. Viruses enter the host through the skin and mucosal surface, where large numbers of microorganisms are colonized (Schmidt et al., 2018; Lunjani et al., 2019; Xu and Li, 2019; Peixoto et al., 2021). Probiotics are critical for the host to inhibit incoming pathogen infections (Lloyd-Price et al., 2016; Piewngam et al., 2018). A large body of literature has shown that probiotics have antiviral effects (Pradhan et al., 2021; Salaris et al., 2021). In the present review, we summarized and discussed the mechanisms of antiviral effects of probiotics, which opens up new perspectives on the use of antiviral strategies, provides new targets for the research and development of antivirals, and helps to provide a clearer background for probiotics-based antivirals.

## Probiotics Contribute to the Chemical Barrier to Exert Antiviral Effects

**Figure 1:**
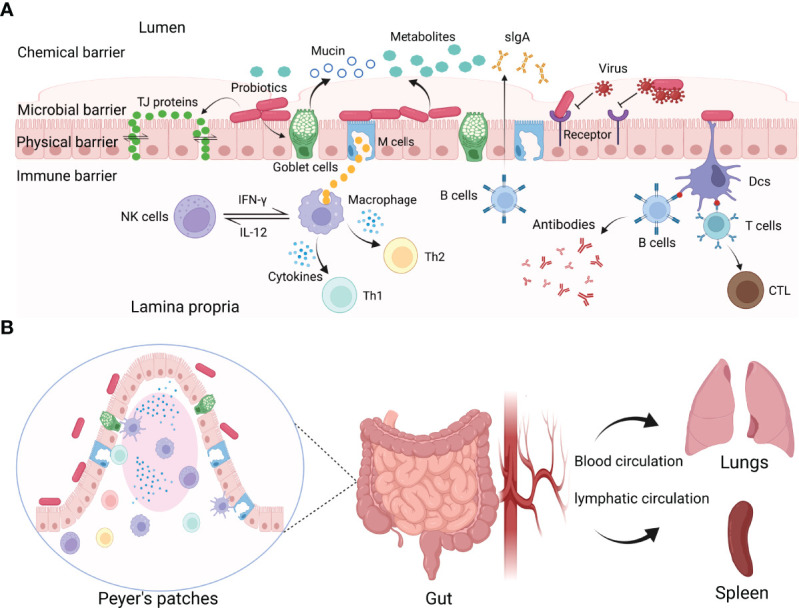
The interactions between probiotics and viruses. **(A)** Probiotics exert protective effects on viral infections by constituting or modulating chemical, microbial, physical, and immune barriers. Probiotics can produce antiviral metabolites, block virus invasion by binding with viruses or competing for the entry receptors, regulating the tight junctions of IECs, and modulating mucosal immune responses. **(B)** Probiotics modulate systemic immune responses.

The chemical barrier is the first barrier that pathogenic microorganisms encounter after they invade the intestine. Pathogens must break through the chemical barrier before they get access to the epithelium. Probiotics utilize carbohydrates in the host to produce various metabolites, including antimicrobial peptides (AMPs), hydrogen peroxide (H_2_O_2_), lactic acid, short-chain fatty acids (SCFAs), and extracellular vesicles (EV) (Knezevic et al., 2005; Gosmann et al., 2017; Vieira et al., 2017; Tyssen et al., 2018; Nahui et al., 2019). It has been revealed that the metabolites released by probiotics display important protective activities to inhibit viral infections. These metabolites form a micro-environment that is not conducive to viral reproduction (**Figure 1A**).

AMPs are known as short, positively charged, and amphipathic peptides with a broad scope of antimicrobial activity against bacteria, viruses, fungi, and protozoa (Boparai and Sharma, 2020; Bosch and Zasloff, 2021). In recent years, AMPs have attracted interests due to their therapeutic potential. Bacteriocins are the AMPs derived from bacteria (Fry, 2018). While the antibacterial effects of bacteriocins are somewhat deciphered, their antiviral effects remain to be further studied. Enterocin B produced by *Enterococcus* was shown to inhibit the cytopathic effects of influenza virus (H1N1 and H3N2) in Madin-Darby canine kidney cells (Ermolenko et al., 2019). Presumably, bacteriocins could lead to the aggregation of viral particles, block viral particles through binding to the host cell receptors or inhibiting key steps in the viral replication cycle (Wachsman et al., 2003). Bacteriocins are structurally diverse with similar antiviral mechanisms.

The probiotics that play protective roles in the vaginal mucosa are dominated by the *Lactobacillus* genus. Numerous studies have demonstrated that metabolites released by *Lactobacillus* have antiviral effects on human immunodeficiency virus (HIV). H_2_O_2_, one of the metabolites produced by *L. acidophilus*, is toxic to many viruses, including HIV. It is reported that the prevalence of H_2_O_2_-producing *Lactobacilli* was lower in HIV-positive compared with HIV-negative women (Knezevic et al., 2005). Another study showed that H_2_O_2_ can inhibit HIV replication in CEM (a human T-lymphoblastic cell line) cells (Klebanoff and Coombs, 1991). The H_2_O_2_-producing probiotics present in the vagina of healthy women have been suggested as some bacteria that maintain health. Disruption of HIV by the probiotics is caused by the toxicity of H_2_O_2_. Notably, there are significant differences in H_2_O_2_ production among different *Lactobacilli* strains, and the same strain may not even produce H_2_O_2_ by changing the culture conditions.

Previous studies on the antiviral effects of lactobacillus-based probiotics have focused on H_2_O_2_, but recent studies have shown that lactic acid is a major antiviral factor produced by *Lactobacilli* in the vaginal mucosa (O'Hanlon et al., 2011; Liu et al., 2021). Lactic acid, a final product of carbohydrates, is an important metabolite of *Lactobacillus* with anti-HIV activity (Gosmann et al., 2017; Nahui et al., 2017; Tyssen et al., 2018). The mechanism of anti-HIV effects of lactic acid-producing bacteria is likely multifactorial. First of all, lactic acid may bind to the viruses and affect the function of the virus, thereby restricting the viruses from invading host cells. Secondly, lactic acid may disrupt the viral envelope and lyse the virions of enveloped viruses (Tachedjian et al., 2017). Besides HIV, lactic acid also has a significant inhibitory effect on herpes simplex virus (HSV) (Conti et al., 2009). It has been confirmed that lactic acid successfully interferes with viral replication in cells and that both the virucidal activity and the inhibition of replication were correlated to acidic pH values.

Microorganisms in the colon produce SCFAs by using dietary carbohydrates as substrates, primarily acetate, propionate, and butyrate, as final products (Wong et al., 2006). The ability to produce SCFAs by both *Lactobacilli* and *Bifidobacteria* is highlighted (LeBlanc et al., 2017). Probiotics have been reported to regulate butyrate metabolism resulting in an enhancement of host resistance to influenza virus infection. The correlation analysis revealed that the butyrate was negatively related to viral loading (Lu et al., 2021). SCFAs produced by probiotics are also potential regulatory effectors of epithelial proliferation in the gut (Wang et al., 2018). In addition, SCFAs can not only act on the gut but also transport to distant sites through blood circulation as a chemical signal for communication. For example, the abundance of SCFAs-producing bacteria and serum SCFA levels increased in respiratory syncytial virus (RSV)-infected mice after administration of a probiotic mixture (LeBlanc et al., 2017). Likewise, similar findings have been reported on antiviral responses induced by commensal microbiota (Antunes et al., 2019).

*Lactobacillus* can produce extracellular vehicles (EVs), along with other bacterial and mammalian cells, which are essential for the communication between bacteria and cells. It has been proved that *L. crispatus* BC3 and *L. gasseri* BC12 protected human cervicovaginal and tonsillar tissues from HIV-1 infection *in vitro* by producing EVs (Nahui et al., 2019). EVs inhibit HIV-1 infection by reducing HIV-1 entry/attachment to target cells. In addition, probiotics can refine the barrier function by promoting goblet cells to secrete mucin to form a mucin layer above the intestinal epithelium, protecting the mucosa from virus attachment (Engevik et al., 2019; Lorella et al., 2020). For instance, probiotics mixture VSL#3 induced mucin expression in colonic epithelial cells and prompted goblet cells to secrete mucin against pathogen attachment (Caballero-Franco et al., 2007). Relevant studies have confirmed that probiotics can secrete extracellular proteins, weaken the adhesion of pathogens, and protect the intestinal cells (Liu et al., 2020). Viruses are intracellular pathogens that require the host machinery to replicate. These metabolites exert antiviral effects by preventing the viruses from attaching to host cells or directly killing the viruses, which is a broad-spectrum antiviral mechanism. In summary, probiotics enhance the chemical barrier to maintain host health by producing antiviral metabolites or stimulating goblet cells to produce mucin.

## Probiotics Constitute the Microbial Barrier to Exert Antiviral Effects

Besides producing some antiviral metabolites, probiotics can also inhibit viruses directly by interacting with viruses or competing for the cellular receptors to inhibit virus entry into host cells (**Figure 1A**) (Lievin-Le and Servin, 2014).

Several studies have revealed that probiotics can inhibit the infection of vesicular stomatitis virus (VSV) *in vitro*. It was suggested that pre-incubation of cell monolayers with probiotics, or probiotics with VSV can decrease VSV titer (Botic et al., 2007). A possible mechanism of inhibiting VSV replication is that probiotics compete with the virus for cell binding and interference with virus attachment or entry. Another possible mechanism is that probiotics can trap VSV specifically or nonspecifically. The above are two possible mechanisms by which probiotics inhibit VSV infection, and more mechanisms remain to be elucidated. *B. subtilisis* is one of the probiotics with excellent antiviral and immune regulation properties. The current study proved that *B. subtilisis* OKB105 inhibited the entry of transmissible gastroenteritis virus (TGEV) into the intestinal porcine epithelial cell line (IPEC-J2) by competing for the entry receptors (Wang et al., 2013). Another study revealed that *Enterococcus faecium* NCIMB 10415 was efficient to inhibit swine influenza virus (SIV) infection by the direct interaction between the SIV and probiotics (Wang et al., 2013). Published literature analysis indicated that the antiviral effects of probiotics are strain-dependent. Besides, the probiotics also limit pathogen access to nutrient resources. Furthermore, studies have revealed that the mucosal immune system of germ-free mice is immature due to the lack of microorganism colonization of their mucosal surfaces (Shen et al., 2014). The colonization of microorganisms also promotes the maturation of the host mucosal immune system. The microbial barrier constituted by probiotics is very important to inhibit viral infections and provides an insight into the development of novel antiviral strategies in the future.

## Probiotics Strengthen the Physical Barrier to Exert Antiviral Effects

The physical barrier is composed of epidermides and mucosae (Karst, 2016). Probiotics form a defensive barrier against the invasion of viruses by enhancing the tight junctions (TJs) between intestinal epithelial cells (IECs) (**Figure 1A**).

TJs are an important form of intercellular connection and the most important structure of the mucosal barrier (Otani and Furuse, 2020). IECs are interconnected by the TJ proteins, which are the membrane protein complex with defensive functions formed between epithelial cells and endothelial cells (Gunzel and Fromm, 2012). Different species of probiotics have been reported to protect the host from viral infections by regulating the production of the TJ proteins (Kanmani and Kim, 2019; Lorella et al., 2020). *Lactiplantibacillus* (*Lp.*) *plantarum*, *Weissella cibaria*, or *Latilactobacillus* (*Ll.*) *sakei* could protect IECs from viral infections by maintaining the activity of TJ proteins. The TJ proteins between epidermal cells are necessary to maintain the integrity of the mucosal barrier. An intact mucosal barrier can prevent pathogens or antigenic elements of pathogens from entering the lamina propria of the gut. Disruption of the epithelial barrier caused by the TJ proteins deficiency will lead to microorganism translocation to the mucosal surface, disrupting microbial homeostasis. Furthermore, it has been confirmed that *Bifidobacterium* (*Bf.*) *breve* treatment promoted the proliferation of small intestine epithelial in mice (Ishizuka et al., 2009). The proliferation of small intestinal epithelial cells promotes the production of new cells and the shedding of necrotic cells. The physical barrier created by the epithelial cells constantly renews itself and enhances the ability of the mucosa physical barrier to resist pathogens. Moreover, probiotics modulated the trans-epithelial electrical resistance and epithelial permeability (Krishnan et al., 2016). The balance of the intestinal microbial environment is necessary for humans and animals to maintain homeostasis. In summary, probiotics can inhibit viral infections by maintaining intestinal permeability and mucosal barrier integrity.

## Probiotics Modulate the Immune Barrier to Exert Antiviral Effects

One of the most beneficial effects of probiotics is to modulate the immune response (Shida and Nanno, 2008; Azad et al., 2018). The immune barrier is the last line of the host to inhibit viral infections, consisting of mucosal and systemic immune responses (**Figures 1A, B**) (Ashraf and Shah, 2014; La Fata et al., 2018).

### Mucosal Immune Responses

Generally, there are large numbers of lymphoid tissues and immune cells in the gastrointestinal tract than in the rest of the body combined. The mucosal immune system (MIS), also known as mucosa-associated lymphoid tissues (MALTs), is mainly composed of immune tissues, cells, and molecules. The mucosa is directly connected to the external environment and involved in complex immune responses (Pelaseyed et al., 2014). Various immune cells are engaged in innate immune response. Natural killer (NK) cells are pivotal members of innate immunity that play key roles in recognizing and killing target cells and regulating immune responses. Activation and proliferation of NK cells can limit viral replication effectively. It has been reported that *Lp. plantarum* 06CC2 reduces HSV-1 virus yields in the brain of mice (Matsusaki et al., 2016). *Lp. plantarum* 06CC2 enhanced immunomodulatory activity by increasing the mRNA expression of IL-12 and IFN-γ in Peyer’s patches (PPs). IL-12 and IFN-γ can activate NK cells and macrophages effectively. Macrophages and NK cells cooperate with each other for virus clearance.

Pattern recognition receptors (PRRs) have extensive interactions between microorganisms and hosts. PRRs are specialized molecules that host cells recognize pathogens. At present, PRRs mainly include Toll-like receptors (TLRs), nucleotide-binding oligomerization domain (NOD)-like receptors (NLRs), retinoic acid-inducible-like receptors (RLRs), C-type lectin receptors (CLRs), absent in melanoma 2 (AIM2)-like receptors (ALRs) and cyclic GMP-AMP synthase (CGAs). Members of the TLRs can identify a wide range of pathogens, such as bacteria, viruses, and fungi. It has been demonstrated that *Lacticaseibacillus rhamnosus* GG (LGG) is therapeutically effective on diarrhea induced by rotavirus (RV) infection by the TLR3 signaling pathway (Aoki-Yoshida et al., 2016). *Ligilactobacillus (Lg.) salivarius* FFIG35 and FFIG58 also displayed antiviral effects by activating the TLR3 signaling pathway (Indo et al., 2021). TLR3 mainly recognizes nucleic acids in endosomes. Activation of TLR3 increases the expression of type I IFNs. In addition to activating TLR3, probiotics can also activate other PRRs. For example, *L. acidophilus* could enhance the antiviral effects by inducing the expression of virus immune defense genes in dendritic cells (DCs) in mice (Weiss et al., 2010). The expression of immune defense genes induced by viruses was dependent on the activation of the TLR2 pathway. In addition, *Lp. plantarum* could inhibit pneumovirus (PMV) infection in mice *via* the NLR (NOD2) and TLR (TLR2) pathways (Rice et al., 2016). Both NOD2 and TLR2 mediate the important innate immune response to inhibit viral infections. Several studies have shown that the probiotic mixtures also have immunomodulatory activity. The probiotics mixture (*L. helveticus* R0052, *Bifidobacterium* R0033, and *Bifidobacterium* R0071) had a major impact on downregulating the expression of proinflammatory cytokines, such as IL-6, IL-8, and IL-1β (Macpherson et al., 2014). The major effects include the upregulated expression of TLR3, mitogen-activated protein kinase, and factor-kappa B (NF-κB) expression. Furthermore, *Lp. plantarum*, *W. cibaria*, and *Ll. sakei* could modulate innate antiviral immune responses induced by poly(I:C) in IECs by activating the TLRs and NF-κB pathways (Kanmani and Kim, 2019). The NF-κB signaling pathway is associated with the production of proinflammatory cytokines. During viral infections, activating the NF-κB signaling pathway can increase the production of proinflammatory cytokines and regulate the function of immune cells.

IFNs are important mediators of antiviral immunity and regulation of immune system homeostasis (Stefan et al., 2020). *Lc. rhamnosus* CRL1505 has been reported to inhibit viral infections by inducing type I IFNs in intestinal antigen-presenting cells (APCs) (Villena et al., 2014). *Lc. paracasei* DG can also significantly induce the expression of type I IFNs, which can limit the viral replication and assembly (Ishizuka et al., 2016). Besides, *Lp. plantarum* Lp-1 has been shown to exert an anti-TGEV effect on IPEC-J2 cells by inducing large amounts of IFN-β in the early stage and activating the JAK-STAT1 pathway in the late stage, and the activated JAK-STAT1 pathway increases the transcription and expression of some antiviral proteins (Wang et al., 2019).

Efficient clearance of viruses depends on the orchestration of innate and adaptive immune responses. Accumulating evidence showed that probiotics can protect the host from viral infections by stimulating an adaptive immune response. *Limosilactobacillus* (*Lm.*) *reuteri* significantly reduced the viral loads of PCV2 in feces, ileum, and mesenteric lymph nodes (MLNs) and increased the immunoglobulin A (IgA) in the ileum (Karaffova et al., 2017). IgA, the main component of the mucosal immune system, is widely distributed in the mucosal secretions of host mucosal tissues, which can inhibit viral attachment to epithelial cells, slow down viral replication, and play important roles in the immune barrier. Increasing evidence has shown that not only live probiotics but also heat-killed probiotics have immunomodulatory effects. For example, heat-killed *Lc. casei* DK128 has broad protection against IFV infection by intranasal treatment by inducing virus-specific antibodies (Jung et al., 2017). Secretory IgA can bind to the virus specifically and the antibody-captured virus can be destroyed by phagocytes.

### Systemic Immune Responses

Probiotics colonized on mucosal surfaces also have distal protective activity against viral infections (**Figure 1B**). It has been confirmed that *Lm. reuteri* has antiviral effects by regulating local mucosal immunity (Karaffova et al., 2017). A recent study has shown that *Lm. reuteri* can also protect the mice from viral infections by regulating the systemic immune responses (Mudronova et al., 2018). Furthermore, orally administrated *Lc. rhamnosus* LA68 induces the expression of some cytokines in the spleen or blood and activates Th1-type immune response (Dimitrijevic et al., 2014). Probiotics or their metabolites can activate immune cells to move distal locations to mediate antiviral responses, or by stimulating immune cells to release cytokines that act distally through blood and lymphatic circulation.

*Bifidobacterium*, one of the main members of intestinal microorganisms, has immunomodulatory activity. *Bf. longum* improved clinical symptoms and reduced mortality in mice after being inoculated intranasally with IFV. The *Bf. longum* MM-2 enhanced NK cell activity in the lungs and spleen and increased the expression of cytokines (IFN-γ, IL-2, IL-12, and IL-18) (Kawahara et al., 2015). *E. faecalis* CECT7121 induced strong activation of DCs and secretion of high levels of inflammatory cytokines (IL-12, IL-6, TNF-α, and IL-10) (Molina et al., 2015). These cytokines are essential for antiviral innate immune response. IL-10 is a well-established inflammatory and immunosuppressive factor, which can regulate immune responses. Due to viral infections, the increase of IL-10 expression can reduce the damage to the host caused by inflammation. DCs are the sentinel cells in the immune system, acting as APCs. They are also the bridge between innate immunity and adaptive immunity and can activate T and B cells directly or migrate to the mesenteric lymph nodes. Besides, orally administrated *Lp. plantarum* conferred protective activities to IFV by producing high levels of IL-12 and IFN-γ in the lungs (Takeda et al., 2011; Park et al., 2013). IL-12 is a key regulatory molecule of innate immunity and adaptive immunity.

IFNs are mediators of innate immunity and play key roles in resisting viral infections. It is reported that double-stranded RNAs produced by lactic acid bacteria (LABs) triggered DCs to produce IFN-β (Kawashima et al., 2018). *Lc. lactis* JCM5805 regulated immune response to IFV in humans by increasing the expression of IFN-α and ISGs (Sugimura et al., 2015). In addition, oral administration of heat-killed *Lp. plantarum* had a protective activity on H1N1 influenza virus infection in mice (Maeda et al., 2009). The beneficial effects of heat-killed *Lp. plantarum* was mediated by inducing host cells to produce type I IFNs. The probiotic mixture can also protect the host from RSV infection by stimulating AM to produce IFNs (Ji et al., 2021). Activation of the IFN signaling pathway increases the expression of ISGs, the major antiviral effectors of IFNs. A growing number of studies have shown that ISGs target different stages of viral replication to inhibit viral infections. It has been reported that *B. velezensis* can reduce the pigeon circovirus (PiCV) viral load significantly in the feces and spleen of pigeons by upregulating Mx1 and signal transducers and activators of transcription 1 (STAT1) genes (Tsai et al., 2021). Myxovirus resistance 1 (*Mx1*), one of ISGs, can block the early transcription of its nucleic acid after the virus invades the cell (Zurcher et al., 1992). Orally administrated *Lc. rhamnosus* can improve the resistance to RSV infection by producing type I IFNs and ISGs (including *IFNAR1, Mx2, OAS1, OAS2*, *RNase L*, and *IFITM3*) (Garcia-Castillo et al., 2020). These molecules enhanced the ability to inhibit RSV infection in mice. *L. gasseri* SBT2055 also has prophylactic potential to prevent RSV infection by upregulating the expression of IFNs and ISGs (Eguchi et al., 2019). Moreover, a candidate protein SRCAP of *L. gasseri* SBT2055 with RSV antiviral activity was identified. But the exact function of SRCAP protein to inhibit RSV replication needs to be further determined. Furthermore, *L. gasseri* SBT2055 can also protect mice from IFV infection by increasing the expression of the *Mx1* and *Oas1a* genes, which are critical for reducing virus titer in the lungs (Nakayama et al., 2014). Upregulated ISGs can inhibit IFV infection effectively.

Moreover, heat-killed *E. faecalis* protected mice suppress influenza virus and enterovirus infections (Chen et al., 2017). The protective activity of *E. faecalis* is associated with the activation of the MCP-1/CCR2 pathway, which might act as a key mediator in the improved antiviral immune response. The expression level of MCP-1 was negatively correlated with virus load.

It is well known that the efficient elimination of viruses relies on adaptive immunity. The mice treated with *Bf. bifidum* produced antibodies, IL-4, IL-12, and IFN-γ, and protected from the challenge with H1N1 influenza virus (Mahooti et al., 2019). IL-4 can induce Th2 immune responses, while IFN-γ modulates Th1 immune responses. The balance between Th1 and Th2 is important for the homeostasis of the host immune system. Oral administration of *Lp. plantarum* (YU) induced sIgA and neutralizing antibodies in bronchoalveolar lavage fluids and suppressed viral proliferation in the lungs (Kawashima et al., 2011). Moreover, oral administration of *Lc. rhamnosus* M21 increased the survival of mice after IFV challenge and sIgA and Th1 cytokines (IL-2 and IFN-γ) were significantly increased (Song et al., 2016). Therefore, the resistance of mice to IFV infection is attributed to the cellular immune responses activated by *Lc. rhamnosus* M21.

## Conclusions and Perspectives

The world is now facing a multitude of novel infectious diseases. Among them, viral diseases are particularly serious. Currently, WHO has stated that severe coronavirus disease 2019 (COVID-19) is a pandemic challenge to humanity. Vaccination is an important tool to inhibit viral infections. But there is a lag in vaccine development for novel viruses and some antiviral drugs also have some adverse reactions. The development of novel antiviral strategies is imminent. The role of probiotics in human and animal health has been an interesting topic in recent years. Probiotics have been proved to exert antiviral effects, representing safe alternative prophylactics for viral diseases in the future (Stavropoulou and Bezirtzoglou, 2020; Sundararaman et al., 2020). However, only a few probiotics have been investigated for their antiviral effects, and the clinical data are still insufficient. At present, the antiviral effects of probiotics were mainly carried out *in vitro* or in mouse models, which needs to be validated in the natural host of the virus. More animal models remain to be established. Accumulating evidence has indicated that the antiviral effects of probiotics are correlated with the routes of administration. The usual delivery route for probiotics is oral administration. But recent studies have shown that probiotics administered intranasally or sublingually can also reduce viral loads and improve the survival of animals. Intranasal and sublingual routes may be alternatives to oral administration. The dosage of probiotics will also affect the antiviral effects. The same probiotics strain in different doses can induce different immune responses. An excessive dose of probiotics may increase the risk of immunosuppression. Thus, we should pay more attention to the species, dosages, and routes of administration of probiotics when using probiotics-based antiviral agents. In addition, more clinical studies should be conducted to reveal which probiotics or their combinations would be the most effective ones for specific viruses.

Homeostasis of the immune system helps the host against viral infections. It has been proved that probiotics can activate not only local immune response but also systemic immune response to viral infections. The exact mechanisms remain unverified. Though the antiviral mechanism of probiotics has been partially uncovered, including probiotic components or metabolites and corresponding host PRRs contributing to the antiviral effects as shown in **Table 1**, it is necessary to understand the antiviral effects of probiotics in more details and more extensive and accurate investigations are demanded to clarify the mechanisms underlying the antiviral effects of probiotics using novel molecular tools and technologies. Understanding the antiviral immunity of humans and animals in the context of probiotics is conducive to the development of new antiviral strategies. The antiviral effects of various probiotics will become an interesting area of future research.

**Table 1:** Antiviral effects and underlying mechanisms of various probiotics.

Probiotics	Tested virus	Models	Mechanisms	References
*Bf. longum* MM-2	H1N1	Mouse	Enhancing NK cells activities; Increasing the expression of cytokines (IFN-γ, IL-2, IL-12, and IL-18).	Kawahara et al., 2015
*Lp. plantarum* 06CC2	H1N1	Mouse	Increasing the expression of cytokines (IL-12 and IFN-γ).	Takeda et al., 2011
Heat-killed *Lc casei* DK128	H3N2	Mouse	Increasing the number of AM; Inducing virus-specific antibodies; Reducing the expression of proinflammatory cytokines.	Jung et al., 2017
*Bf. bifidum*	H1N1	Mouse	Inducing virus-specific antibodies; Increasing the expression of cytokines (IL-4 and IFN-γ); Enhancing lymphocyte proliferative responses.	Mahooti et al., 2019
*E. faecium* NCIMB 10415	SIV	3D4/21 and MDBK cell	Direct interaction with viruses.	Wang et al., 2013
*B. clausii*	RV	Caco-2 cells	Increasing the expression of mucin 5AC and TJ proteins.	Lorella et al., 2020
*L. rhamnosus GG* (LGG)	RV	Mouse	Activating the TLR3 pathway.	Aoki-Yoshida et al., 2016
*Lg. salivarius* FFIG35 and FFIG58	RV	PIEs	Activating the TLR3 pathway.	Indo et al., 2021
*Bf.* MCC12 and MCC1274	RV	PIEs	Activating the NF-κB signaling pathway; Increasing the expression of IFN-β; Increasing the expression of ISGs.	Ishizuka et al., 2016
*Lactobacillus acidophilus* (LB^+^)	HIV	CEM cells	Producing H_2_O_2_.	Klebanoff and Coombs, 1991
*L. crispatus* BC3 and *L. gasseri* BC12	HIV	CD4^+^ T cell lines, MT-4 and Jurkat; Human cervix, vaginal and tonsillar tissues *in vitro*.	Reducing virus entry/attachment to target cells.	Nahui et al., 2019
*B. subtilisis* OKB105	TGEV	IPEC-J2 cells	Competing with entry receptors.	Wang et al., 2013
*Lp. plantarum* Lp-1	TGEV	IPEC-J2 cells	Increasing the expression of IFN-β; Activating the JAK-STAT1 pathway.	Wang et al., 2019
*Ll. reuteri*	PCV2	Mouse	Increasing the expression of cytokines (chemokines, IFN-γ, and IgA).	Karaffova et al., 2017
*Ll. reuteri*	PCV2	Mouse	Increasing the percentage of CD8^+^ and CD49b^+^CD8^-^ cells; Increasing the expression of cytokines (RANTES, GM-CSF, IFN-γ, and IgA).	Mudronova et al., 2018
*B. velezensis*	PiCV	Pigeon	Increasing the expression of cytokines (IFN-γ, Mx1, STAT1, TLR2, and TLR4).	Tsai et al., 2021
*Lc. rhamnosus* CRL1505	RSV	Mouse	Increasing the expression of cytokines (IFN-α, IFN-β, IFN-γ, and ISGs).	Villena et al., 2014
*L. gasseri* SBT2055	RSV	Mouse	Increasing the expression of ISGs.	Eguchi et al., 2019
Probiotic mixture (*L. rhamnosus* GG, *Escherichia coli* Nissle 1917 and VSL#3)	RSV	Mouse	Increasing the expression of IFNs; Restoring of gut microbiota balance.	Krishnan et al., 2016
*Lp. plantarum*	PMV	Mouse	Activating the NOD2 and TLR2 pathways.	Rice et al., 2016

## Author Contributions

YW wrote the manuscript. AM, JH, YS and H-JQ revised this manuscript. All authors contributed to the article and approved the submitted version.

## Funding

This study was supported by the National Key R&D Program of China (Grant no. 2021YFD1801403).

## Conflict of Interest

The authors declare that the research was conducted in the absence of any commercial or financial relationships that could be construed as a potential conflict of interest.

## Publisher’s Note

All claims expressed in this article are solely those of the authors and do not necessarily represent those of their affiliated organizations, or those of the publisher, the editors and the reviewers. Any product that may be evaluated in this article, or claim that may be made by its manufacturer, is not guaranteed or endorsed by the publisher.
